# A Retrospective Review of Outcomes in Intensive Care Unit Patients Infected With SARS-Cov2 in Correlation to Admission Acute Physiologic Assessment and Chronic Health Evaluation II Scores

**DOI:** 10.7759/cureus.14051

**Published:** 2021-03-23

**Authors:** Pratishtha Singh, Kayle Warren, Hannah Adler, Andrew Mangano, Jilian Sansbury, Richard Duff

**Affiliations:** 1 Internal Medicine, Grand Strand Medical Center, Myrtle Beach, USA; 2 Pulmonary and Critical Care Medicine, Grand Strand Medical Center, Myrtle Beach, USA

**Keywords:** covid 19, apache-ii score, continuous renal replacement therapy (crrt), acute renal failure and hemodialysis in icu, venous thromboembolism (vte)

## Abstract

Introduction

Coronavirus disease 2019 (COVID-19) has emerged as a global pandemic that has placed an unprecedented burden on intensive care services worldwide. Identification of a reliable risk-stratification tool for COVID-19 patients is necessary for appropriate resource allocation, selection of clinical management pathways, and guidance of goals of care conversations with families and caregivers in the critical care setting. The Acute Physiologic Assessment and Chronic Health Evaluation (APACHE) II scoring system is one of several predictive models used to classify illness severity and estimate mortality risk on admission to the intensive care unit (ICU). Our retrospective study sought to evaluate the prognostic ability of the APACHE II score in COVID-19 patients according to endpoints of mortality and length of stay (LOS) as well as unfavorable clinical outcomes, including development of acute renal failure (ARF) requiring renal replacement therapy (RRT) and acute venous thromboembolic events (VTE).

Methods

This multicenter retrospective cohort study evaluated a randomized sample of 3,102 patients with confirmed COVID-19 disease admitted to the ICU from January 2020 to May 2020. A total of 395 patients with complete data points for appropriate APACHE II score calculation, absence of the preexisting comorbidities end-stage renal disease, and history of VTE were included. Linear and logistic regression models were employed to evaluate primary outcomes of mortality and LOS as well as secondary outcomes of VTE and ARF requiring continuous renal replacement therapy (CRRT) or hemodialysis (HD).

Key results

Among the 395 patients enrolled, total percent mortality and mean LOS were 37.0% and 12.92 days, respectively. Primary outcome analysis revealed a statistically significant increase in odds of mortality as well as in mean LOS with every additional point increase in APACHE II score from a baseline of zero. Specifically, for every point increase in the APACHE II score, odds of mortality increased by 12% (p value < 0.001), and average LOS increased by 0.2 days (p value < 0.001). In our secondary outcome analysis, 14.43% and 62.2% of the total sample population developed ARF requiring RRT and VTE, respectively. For every additional point increase in APACHE II score from a baseline of zero, odds of requiring CRRT or HD increased by 10% on average (95% CI (1.06, 1.15); p value < 0.001). Similarly, for every additional point increase in the APACHE II score from a baseline of zero, there was a corresponding increase in odds of VTE by 19% (95% CI (1.14, 1.24); p value < 0.001).

Conclusions

The APACHE II score is an effective predictive model of in-hospital mortality and unfavorable clinical outcomes, including prolonged LOS, ARF requiring CRRT or HD, and development of VTE. As therapeutic interventions for COVID-19 evolve, application of this risk-stratification tool may guide clinical management decisions in the critical care setting.

## Introduction

Coronavirus disease 2019 (COVID-19) is a viral disease spectrum, varying from asymptomatic infection to severe acute respiratory distress syndrome, caused by a highly pathogenic enveloped, single-stranded, positive-sense RNA virus of the Coronaviridae family [1]. Identification of COVID-19 patients requiring early and aggressive intervention remains poorly defined and highly controversial. The Acute Physiologic Assessment and Chronic Health Evaluation (APACHE) II score is one of several predictive models used to classify illness severity and estimate mortality risk in critically ill patients [1]. The APACHE II scoring system generates a point score ranging from 0 to 71. It takes into account patient age, comorbidity, and multiple objective measures, including temperature, mean arterial pressure, pH, heart rate, respiratory rate, sodium, potassium, creatinine, hematocrit, white blood cell count, Glasgow Coma Scale, and FiO2 [1]. This risk-stratification tool was not designed for serial calculation, nor was it intended to direct medical management; rather it was evaluated as a tool to predict mortality through the classification of illness severity [1]. Accordingly, this multicenter retrospective cohort study used the APACHE II scoring system to establish baseline mortality risk of patients with COVID-19 admitted to the intensive care unit (ICU).

Given the rapidly evolving landscape of the COVID-19 pandemic, clinically relevant outcomes beyond mortality remain poorly understood. Acute renal failure (ARF) requiring continuous renal replacement therapy (CRRT) or hemodialysis (HD) as well as hypercoagulability evidenced by venous thromboembolic events (VTE) have been well-documented in various case reports since the emergence of COVID-19. Consequently, we sought to further evaluate the burden of ARF requiring CRRT or HD as well as development of VTE in this patient population to define the role of the APACHE II score in predicting these unfavorable clinical outcomes.

## Materials and methods

Study design

This retrospective cohort study evaluated patient information from hospitalized patients in the ICU with confirmed severe acute respiratory syndrome coronavirus 2 (SARS-CoV2) infection. The data was obtained from 59 hospitals using HCA’s retrospective data bank (EDW), which included inpatient laboratory and pharmacy claims coded with International Classification of Diseases (ICD) revision 10. This study was conducted in compliance with the HCA requirements, received institutional review board exemption determination letter through Centralized Algorithms for Research Rules on IRB Exemptions (CARRIE).

Study population

Our cohort was developed using 395 patients admitted to the ICU with confirmed SARS-CoV2 infection from January 2020 to May 2020. A total of 59 US hospitals were in the study. The study index date was defined as the date of initial admission to the ICU, and patients were followed to discharge or death. At baseline, for each patient, demographic, comorbid, clinical, and pharmacy data were extracted. Demographic and clinical characteristics included age, sex, race, and ethnicity. To determine baseline comorbid conditions, we used ICD-10 codes for coronary artery disease (CAD), chronic obstructive pulmonary disease (COPD), heart failure, atrial fibrillation, hypertension (HTN), diabetes mellitus (DM), peripheral vascular disease (PVD), tobacco abuse, alcohol abuse, and chronic kidney disease (CKD). Vital signs such as data of temperature, mean arterial pressure, heart rate, respiratory rate, Glasgow Coma Scale, and other measures required for calculation of the APACHE II score were collected. Laboratory data were extracted for each patient and included pH, sodium, potassium, creatinine, blood urea nitrogen, hematocrit, erythrocytes, platelets, white blood cells, FiO2, and an alveolar-arterial (A-a) gradient. Patients with preexisting end-stage renal disease (ESRD), already on HD, or with a history of chronic deep vein thrombosis/pulmonary embolism (DVT/PE) were excluded from the data set.

Outcomes and exposure coding

Patients were categorized based on their initial APACHE II score. Scores were categorized into seven groups: 5-9, 10-14, 15-19, 20-24, 25-29, 30-34, and >34. The primary outcomes were mortality and length of stay (LOS). The secondary outcome assessed inflammatory markers and their association with APACHE II scores, development of ARF requiring CRRT or HD, and development of acute venous thromboembolism (VTE).

Statistical analysis

A logistic regression model was used to assess the relationship between APACHE II score, mortality, and secondary outcomes of CRRT/HD and development of VTE while controlling for sex, race, and ethnicity. Coefficient estimates and odds ratio were used to assess and read primary and secondary outcomes. These models do not in any way imply causality.

## Results

Our cohort was developed using 395 patients admitted to the ICU with confirmed SARS-CoV2 infection from January 2020 to May 2020. Table 1 shows the study baseline population. Inclusion criteria were all patients aged 18 years and above who were admitted with documented SARS-CoV2 infections. All patients with baseline ESRD requiring dialysis or history of VTE on admission were excluded. All patients in which the entire APACHE II score could not be calculated were excluded. Our cohort evolved from a randomized sample of 3,102 patients with confirmed COVID-19 disease; 2,707 patients were excluded due to the absence of valid and complete vital signs, laboratory values, or Glasgow Coma Scale scores necessary to calculate the APACHE II score or as a result of meeting exclusion criteria of the preexisting comorbidity of ESRD or history of VTE.

**Table 1:** Demographics and Clinical Characteristics This table shows the demographic and clinical characteristics of our cohort, including age, sex, race, and ethnicity, as well as preexisting comorbid conditions, of the 395 patients admitted to the intensive care unit with confirmed COVID-19 disease.

Patient Attributes	APACHE II Score: 5-9	APACHE II Score: 10-14	APACHE II Score: 15-19	APACHE II Score: 20-24	APACHE II Score: 25-29	APACHE II Score: 30-34	APACHE II Score: Greater Than 34
Age							
Minimum	30	20	19	19	36	23	39
Mean (95% Confidence Interval)	45.86 (37.66, 54.05)	54.17 (49.88, 58.46)	60.29 (55.37, 65.22)	68.74 (64.93, 72.55)	68.63 (65.18, 72.09)	71.05 (67.22, 74.87)	69.33 (66.81, 71.85)
Standard Deviation	8.86	13.77	18.73	16.35	13.95	15.32	11.75
Median	48	54.5	60.5	72	70	74.5	69.5
Maximum	55	90	90	90	90	90	90
Race							
Asian	0 (0%)	2 (4.8%)	1 (1.7%)	3 (4.1%)	2 (3.1%)	4 (6.3%)	4 (4.7%)
Black	4 (57.1%)	5 (11.9%)	11 (19%)	11 (15.1%)	10 (15.4%)	20 (31.3%)	21 (24.4%)
Other/Unknown	0 (0%)	18 (42.9%)	20 (34.5%)	18 (24.7%)	11 (16.9%)	11 (17.2%)	27 (31.4%)
White	3 (42.9%)	17 (40.5%)	26 (44.8%)	41 (56.2%)	42 (64.6%)	29 (45.3%)	34 (39.5%)
Ethnicity							
Hispanic	1 (14.3%)	21 (50%)	20 (34.5%)	20 (27.4%)	6 (9.2%)	9 (14.1%)	24 (27.9%)
Non-Hispanic	6 (85.7%)	20 (47.6%)	35 (60.3%)	51 (69.9%)	59 (90.8%)	51 (79.7%)	59 (68.6%)
Other or Declined to Specify	0 (0%)	1 (2.4%)	3 (5.2%)	2 (2.7%)	0 (%)	4 (6.3%)	3 (3.5%)
Sex							
Female	5 (71.4%)	20 (47.6%)	28 (48.3%)	32 (43.8%)	27 (41.5%)	20 (31.3%)	32 (37.2%)
Male	2 (28.6%)	22 (52.4%)	30 (51.7%)	41 (56.2%)	38 (58.5%)	44 (68.8%)	54 (62.8%)
Atrial Fibrillation	0 (0.00%)	3 (7.14%)	7 (12.07%)	20 (27.40%)	21 (32.31%)	22 (34.38%)	37 (43.02%)
Hypertension	6 (85.71%)	21 (50.00%)	29 (50.00%)	34 (46.58%)	22 (33.85%)	20 (31.25%)	25 (29.07%)
Type 2 Diabetes Mellitus	3 (42.86%)	13 (30.95%)	18 (31.03%)	22 (30.14%)	26 (40.00%)	22 (34.38%)	32 (37.21%)
Coronary Artery Disease	1 (14.29%)	3 (7.14%)	8 (13.79%)	17 (23.29%)	20 (30.77%)	18 (28.13%)	20 (23.26%)
Chronic Obstructive Pulmonary Disease	0 (0.00%)	0 (0.00%)	1 (1.72%)	0 (0.00%)	3 (4.62%)	0 (0.00%)	1 (1.16%)
Heart Failure (Systolic or Diastolic)	0 (0.00%)	3 (7.14%)	7 (12.07%)	17 (23.29%)	11 (16.92%)	13 (20.31%)	19 (22.09%)
Chronic Kidney Disease (Stages I Through IV)	1 (14.29%)	0 (0.00%)	8 (13.79%)	15 (20.55%)	20 (30.77%)	26 (40.63%)	37 (43.02%)
Alcohol Abuse	0 (0.00%)	1 (2.38%)	0 (0.00%)	1 (1.37%)	0 (0.00%)	2 (3.13%)	2 (2.33%)
Tobacco Abuse	0 (0.00%)	1 (2.38%)	2 (3.45%)	1 (1.37%)	2 (3.08%)	2 (3.13%)	2 (2.33%)
Peripheral Vascular Disease	0 (0.00%)	0 (0.00%)	1 (1.72%)	1 (1.37%)	3 (4.62%)	3 (4.69%)	2 (2.33%)

Mortality

**Figure 1:**
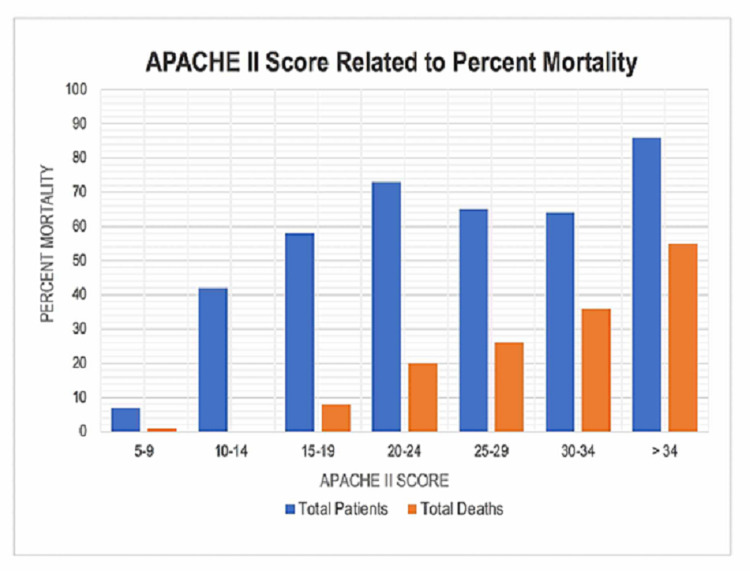
APACHE II Score Related to Percent Mortality This figure shows the relationship between APACHE II score and mortality, while controlling for race, ethnicity, sex, and administration of anticoagulation. APACHE II, Admission Acute Physiologic Assessment and Chronic Health Evaluation II.

Higher APACHE II scores were associated with increased mortality. Out of 395 patients, 146 (37%) patients died (Table 2 and Figure 1).

**Table 2:** Primary and Secondary Outcomes This table shows summary of primary and secondary outcomes according to APACHE II score. Primary endpoints evaluated included mortality and mean length of stay (LOS). Secondary endpoints included venous thromboembolic events (VTE) and acute renal failure requiring continuous renal replacement therapy (CRRT) or hemodialysis (HD).

	APACHE II Score: 5-9	APACHE II Score: 10-14	APACHE II Score: 15-19	APACHE II Score: 20-24	APACHE II Score: 25-29	APACHE II Score: 30-34	APACHE II Score: Greater Than 34
Mortality	1 (14.3%)	0 (0%)	8 (13.8%)	20 (27.4%)	26 (40.0%)	36 (56.3%)	55 (64.0%)
Mean Length of Stay	8.86	11.30	10.10	11.90	14.10	12.30	16.40
Venous Thromboembolism	3 (42.86%)	6 (14.29%)	14 (24.14%)	39 (53.42%)	46 (70.77%)	55 (85.94%)	83 (96.51%)
Continuous Renal Replacement Therapy (CRRT) or Hemodialysis (HD)	0 (0.00%)	0 (0.00%)	1 (1.72%)	5 (6.85%)	8 (12.31%)	14 (21.9%)	29 (33.7%)

**Figure 2:**
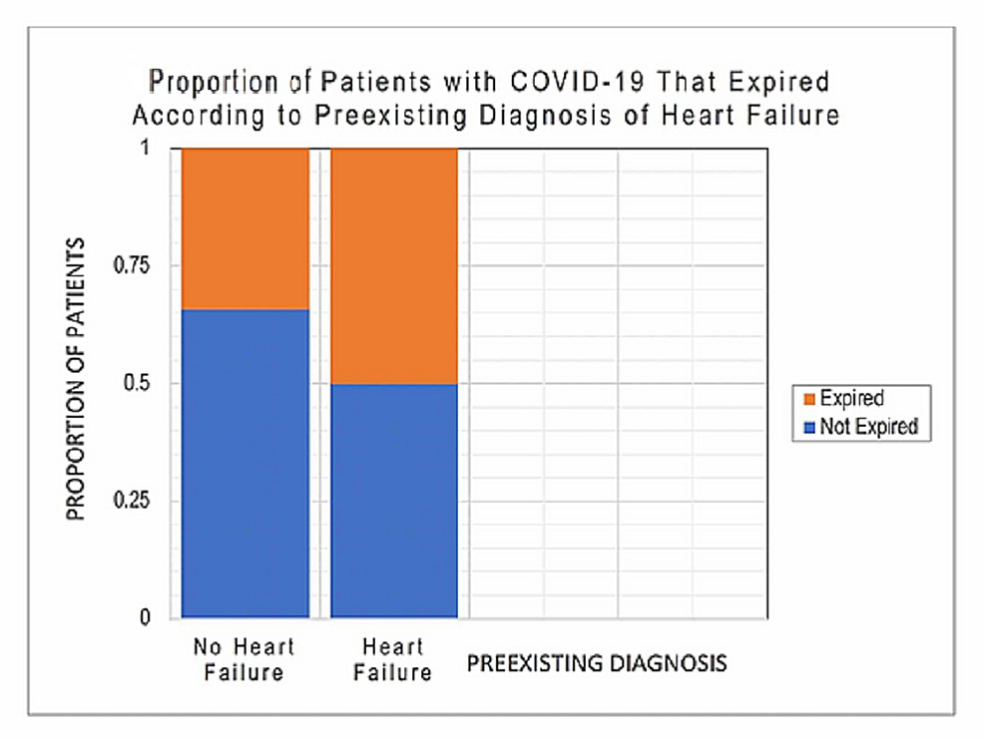
Proportion of Patients With COVID-19 That Expired According to Preexisting Diagnosis of Heart Failure This figure demonstrates the proportion of patients with COVID-19 disease that expired according to presence or absence of preexisting systolic or diastolic congestive heart failure.

Using a logistic regression model for every additional point increase in the APACHE II score above zero, patients' odds of mortality increased on average by 12% (95% CI (0.08, 0.14); p < 0.001). During our primary outcome analysis, an interesting finding revealed a relationship between patients with heart failure and CKD as a documented comorbidity. Although a statistically significant result (α = 0.05) was not observed with a logistic regression model, other univariate analyses were used to see if there were any clinically relevant patterns. A chi-squared test revealed that the relationship between heart failure and patients' mortality is also statistically significant (p = 0.02) suggesting that patients with heart failure had higher mortality in our patient population (Figure 2).

**Figure 3:**
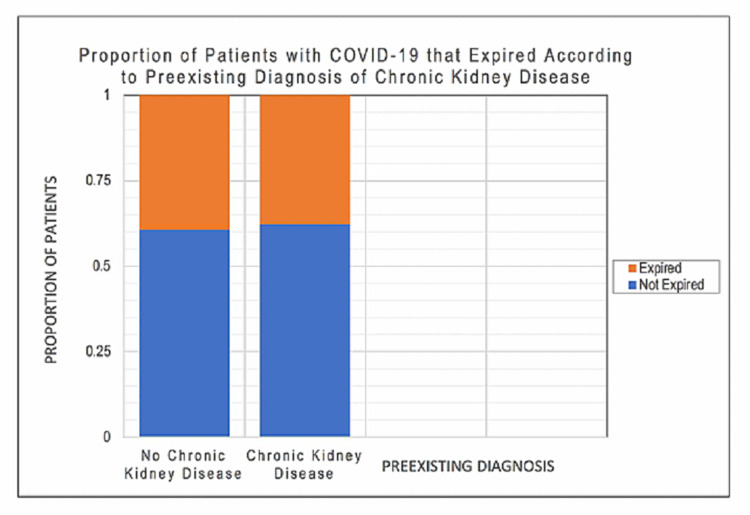
Proportion of Patients With COVID-19 That Expired According to Preexisting Diagnosis of Chronic Kidney Disease This figure demonstrates the proportion of patients with COVID-19 disease that expired according to presence or absence of preexisting chronic kidney disease (Stages I through IV).

According to a chi-squared test, there was a statistically significant relationship between patients with CKD and increased mortality (p < 0.001), suggesting that patients with a prior diagnosis of CKD with COVID-19 infection had higher mortality than those without prior diagnosis of CKD (Figure 3).

Length of stay

A linear regression model revealed that for every additional point in the APACHE II score, there is a corresponding 0.2-day average increase in the patient’s LOS (p < 0.001). Average LOS for different APACHE II scores is noted in Table 2, and for all patients it was noted to be at 12.92 days.

A secondary outcome reviewed APACHE II score and development of ARF requiring CRRT or HD treatment. Out of 395 total patients, 57 patients (14.43%) developed ARF requiring CRRT/HD (Table 2). After controlling for all other variables in a logistic regression analysis model, for every additional point in the APACHE II score from a baseline of zero, patients’ odds of getting CRRT or HD increased 10% on average (95% CI (1.06, 1.15); p < 0.001) (Table 3). After controlling for all other variables in the model, patients with an indicated race of “black” had 3.41 times higher odds of requiring CRRT/HD (95% CI (1.53, 7.77); p = 0.003) on average than patients with an indicated race of “white.” Patients who indicated race of “other/unknown” had 3.38 times higher odds of requiring CRRT/HD than patients with an indicated race of “white” (95% CI (1.12, 10.43), p = 0.03) (Table 3). Controlling for all other variables in the model, patients with an indicated sex of “female” had 55% lower odds of requiring CRRT/HD than patients with an indicated sex of “male” (95% CI (0.22, 0.93); p = 0.04). Patients with a history of atrial fibrillation had 2.02 times higher odds of requiring CRRT/HD than those without a history of atrial fibrillation (95% CI (1.01, 4.07), p = 0.05) (Table 3).

**Table 3:** Multivariate Logistic Regression Analysis Model for APACHE II Score and CRRT/HD This is a table of coefficients from a multivariate logistic regression analysis for APACHE II score and CRRT or HD. APACHE II, Admission Acute Physiologic Assessment and Chronic Health Evaluation II; CRRT, continuous renal replacement therapy; HD, hemodialysis.

Model Term	Coefficient Estimate	Odds Ratio (95% Confidence Interval)	p Value
(Intercept)	-5.18	0.01 (0.00, 0.02)	< 0.001
Apache II Score	0.10	1.10 (1.06, 1.15)	< 0.001
Race = Black	1.23	3.41 (1.53, 7.77)	0.003
Race = Asian	0.32	1.38 (0.17, 7.34)	0.73
Race = Other/Unknown	1.22	3.38 (1.12, 10.43)	0.03
Ethnicity = Hispanic	-0.78	0.46 (0.14, 1.43)	0.19
Ethnicity = Unknown/Decline to Specify	-0.51	0.60 (0.07, 3.48)	0.59
Sex = Female	-0.77	0.46 (0.22, 0.93)	0.04
C-Reactive Protein	0.01	1.01 (0.98, 1.04)	0.62
Atrial Fibrillation	0.71	2.02 (1.01, 4.07)	0.05
Hypertension	-0.57	0.57 (0.24, 1.32)	0.20
Type II Diabetes	0.08	1.08 (0.54, 2.14)	0.82
Cardiovascular Disease	0.27	1.31 (0.61, 2.75)	0.48
Heart Failure	-0.39	0.68 (0.27, 1.63)	0.40
Chronic Kidney Disease	-0.19	0.83 (0.39, 1.73)	0.62
Other Comorbidity*	-0.16	0.86 (0.22, 2.65)	0.80
*Other comorbidities of interest (merged due to low number of encounters): Chronic obstructive pulmonary disease, alcohol abuse, tobacco use, and peripheral vascular disease.			

An additional secondary outcome reviewed APACHE II score and development of acute thromboembolism. From a total of 395 patients analyzed, 246 patients developed acute venous thromboembolism, which accounted for 62.2% of the total patient population (Table 2). After controlling for all other variables, a multivariate logistic regression analysis revealed that for every additional unit increase in the APACHE II score from baseline of zero, there was a corresponding 19% increase in the patients’ odds of having acute thromboembolism (95% CI (1.14, 1.24); p < 0.001) (Table 4). Patients who also indicated their sex as “female” had 57% lower odds of developing thromboembolism than patients who indicated their sex as “male” (95% CI (0.24, 0.77); p < 0.01). Patients with a history of hypertension (HTN) had 2.03 times higher odds of developing acute VTE than patients without a diagnosis of HTN (95% CI (1.03, 4.15); p = 0.04), and patients with a history of CKD had 10.46 times higher odds of developing acute VTE than patients without a diagnosis of CKD (95% CI (4.42, 27.64); p = 0.001). These findings are shown in Table 4.

**Table 4:** A Multivariate Logistic Regression Analysis Model for APACHE II Score and Venous Thromboembolism This is a table of coefficients from a multivariate logistic regression analysis for APACHE II score and venous thromboembolism. APACHE II, Admission Acute Physiologic Assessment and Chronic Health Evaluation II.

Model Term	Coefficient Estimate	Odds Ratio (95% Confidence Interval)	p Value
(Intercept)	-4.63	0.01 (0.00, 0.03)	< 0.001
Apache II score	0.17	1.19 (1.14, 1.24)	< 0.001
Race = Black	0.59	1.80 (0.81, 4.12)	0.16
Race = Asian	0.68	1.96 (0.45, 9.71)	0.38
Race = Other/Unknown	0.06	1.06 (0.44, 2.63)	0.89
Ethnicity = Hispanic	-0.77	0.46 (0.18, 1.15)	0.10
Ethnicity = Unknown/Decline to Specify	-0.33	0.72 (0.13, 3.89)	0.70
Sex = Female	-0.84	0.43 (0.24, 0.77)	0.01
C-Reactive Protein	0.03	1.03 (0.99, 1.06)	0.16
Atrial Fibrillation	-0.44	0.65 (0.30, 1.36)	0.25
Hypertension	0.71	2.04 (1.03, 4.15)	0.04
Type II Diabetes	0.53	1.71 (0.90, 3.27)	0.10
Cardiovascular Disease	0.48	1.61 (0.77, 3.46)	0.21
Heart Failure	0.23	1.26 (0.52, 3.11)	0.61
Chronic Kidney Disease	2.35	10.46 (4.42, 27.64)	< 0.001
Other Comorbidity*	-0.99	0.37 (0.12, 1.16)	0.08
*Other comorbidities of interest (merged due to low number of encounters): Chronic obstructive pulmonary disease, alcohol abuse, tobacco abuse, and peripheral vascular disease.			

## Discussion

The emergence of COVID-19 as a global pandemic has placed an unprecedented burden on intensive care services worldwide as a result of the extraordinary demand for advanced respiratory support strategies, including non-invasive and invasive mechanical ventilation. Initial mortality estimates exceeded 90% in patients undergoing invasive mechanical ventilation; which may reflect resource limitations in the pandemic setting as well as the rapid progression of COVID-19 disease itself [2]. A more recent systematic review and meta-analysis of 24 observational studies, including 10,150 patients from centers across Asia, Europe, and North America, reported a pooled ICU mortality rate of upwards of 40% in COVID-19 patients [3]. This remains disproportionately higher than the 22% mortality rate reported in other viral pneumonias admitted to the ICU, including severe acute respiratory syndrome (SARS) and Middle Eastern respiratory syndrome (MERS) [4,2].

Despite the aforementioned, a reliable predictive model of mortality in COVID-19 disease has yet to be established. A single-center, retrospective study based in Wuhan, China, Hubei Province sought to accurately classify illness severity and estimate mortality risk in patients with COVID-19 using three existing scoring systems, including the Acute Physiologic Assessment and Chronic Health Evaluation (APACHE) II score; Sequential Organ Failure Assessment (SOFA) score; and Confusion, Urea, Respiratory Rate, Blood Pressure, and Age 65 (CURB-65) score [5]. The results of this study demonstrated that the APACHE II score is an effective predictive model of mortality in COVID-19 patients as compared to the SOFA and CURB-65 scores [5]. The above-mentioned findings are likely attributable to the fact that the APACHE II score accounts for both age and presence of existing comorbidities, which appear to be independent predictors of disease severity and mortality risk in COVID-19 [5]. This is in comparison to the CURB-65 score, which accounts for age alone, and the SOFA score, which fails to account for either age or presence of comorbidities [5]. Ultimately, there remains a need for a practical risk-stratification model to accurately predict mortality in COVID-19 disease, particularly in time-sensitive and resource-limited settings such as the ICU.

In this multicenter retrospective cohort study, we sought to further define the prognostic ability of the APACHE II score in COVID-19 patients in the ICU, specifically evaluating for primary endpoints of mortality and LOS. According to our multivariate logistic regression analysis, controlling for race, ethnicity, and sex, the APACHE II score was positively associated with mortality in COVID-19 disease in the critical care setting (p value < 0.001). Moreover, for every additional point obtained in the APACHE II score above zero, mortality increased by 12%, on average. In addition, while controlling for the same demographic variables, for every additional point measured by the APACHE II score, there was a corresponding increase in LOS by approximately 0.20 days (p value: 0.01). Primary outcome analysis of our sample population with comorbidities including hypertension, type 2 diabetes mellitus, CAD, COPD, heart failure, CKD, PVD, and alcohol and tobacco abuse revealed increased mortality in heart failure and CKD patients with COVID-19. Although the above-mentioned was not statistically significant in the initial logistic regression model, subsequent univariate analysis with chi-squared testing demonstrated statistical significance. This finding has been documented in existing literature evaluating the impact of comorbidity on mortality in COVID-19 disease [6-8]. Ultimately, these results suggest that the APACHE II score can reliably predict in-hospital mortality and the LOS in COVID-19 disease, further demonstrating that it is a pragmatic risk-stratification tool for COVID-19 patients on admission to the ICU.

Our secondary outcome analysis sought to evaluate the burden of ARF in critically ill patients with COVID-19 to define the role of the APACHE II score in predicting the need for CRRT or HD. Acute kidney injury is a common complication associated with COVID-19 and is associated with increased morbidity and mortality [9], and literature review of 12 observational and randomized studies found that up to 20% of patients admitted to ICUs may require continuous renal replacement therapy [10]. As indicated in Table 2, 57 of 395 patients (14.43%) in our cohort developed ARF requiring CRRT or HD. Multivariate logistic regression analysis demonstrated a statistically significant association between a higher APACHE II score and subsequent initiation of CRRT or HD. Specifically, the odds of a patient requiring renal replacement therapy (RRT) or HD increased by 10% on average for every additional point obtained in the APACHE II score above zero (p value < 0.001). A prospective cohort study found that prevalence of kidney disease (not ESRD) on admission was associated with an increased in-hospital mortality [11]. Our study reviewed association with APACHE II scores and patients with CKD; however, no statistical significance was noted with CKD as an underlying comorbidity. This would be attributed to our low power.

Hypercoagulability, evidenced by venous, arterial, and catheter-related thrombosis, is well-documented in patients with COVID-19 disease [12]. The proposed pathogenesis of hypercoagulability in COVID-19 appears to be related to endothelial injury via direct cellular invasion and indirect proinflammatory cytokine release as well as abnormal elevation of circulating prothrombotic factors, leading to macro- and microvascular thrombosis [13]. Initial estimates of the incidence of venous thromboembolism in critically ill patients with COVID-19 approached 25% [14]. Subsequent studies, including a multicenter retrospective cohort study in the Netherlands, also demonstrated a similar cumulative incidence of venous thromboembolism of approximately 25%, despite administration of standard-dose thromboprophylaxis during course of hospitalization [15]. Accordingly, treatment pathways employing prophylactic, intermediate, or therapeutic doses of anticoagulation continue to evolve with emerging evidence of COVID-19 disease-related coagulopathy [12]. Our multicenter retrospective cohort study sought to define the burden of acute venous thromboembolism in COVID-19 disease. Of our sample population, 246 of 395 patients (62.2%) had a documented venous thromboembolic event. According to our multivariate logistic regression analysis, controlling for race, ethnicity, and sex, the APACHE II score was positively associated with VTE in patients with COVID-19. Moreover, for every additional point obtained in the APACHE II score, from a baseline of zero, there was a 19% increase in the odds of sustaining a venous thromboembolic event (p value < 0.001). These results appear to support application of the APACHE II score to critically ill patients with COVID-19 disease. Further investigation is necessary to define the role of the APACHE II score as a predictive model of unfavorable clinical outcomes in COVID-19 in order to guide management decisions in the ICU.

Limitations

This multicenter retrospective cohort study evaluated a diverse patient population across 59 unique hospitals in the United States according to specific endpoints not previously studied in COVID-19 disease. A notable limitation of this study was the sample size. Patients without valid or complete documentation of vital signs, Glasgow Coma Scale scores, or laboratory data on admission to the ICU were strictly excluded, given inability to accurately calculate corresponding APACHE II scores. Additionally, marked variability in documentation of ICD-10 codes largely impaired accurate data collection according to critical secondary endpoints, including ARF requiring RRT as well as acute VTE. Furthermore, it was not possible to differentiate between patients receiving prophylactic versus therapeutically dosed anticoagulation in our data sample, inhibiting our ability to evaluate the role of anticoagulation therapy with respect to our prespecified outcomes. Finally, it is necessary to interpret our observational data with caution, in view of the fact that our findings may have been influenced by unmeasured or unidentified confounders.

## Conclusions

We propose that the APACHE II score could be an effective predictive model for unfavorable clinical outcomes in COVID-19 disease. Accurate prediction of mortality as well as disease-related complications with this risk-stratification tool may facilitate appropriate allocation of resources and direct goals of care conversations with family and caregivers on admission to the ICU. Further investigation of the impacts of intervention on mortality, LOS, and other adverse outcomes is warranted, given these findings.
